# O4-3 An evaluation of the Time to Move workplace physical activity intervention

**DOI:** 10.1093/eurpub/ckac094.027

**Published:** 2022-08-29

**Authors:** Kat Ford, Catherine Sharp, Karen Hughes, Mark A Bellis

**Affiliations:** 1 Medical and Health Sciences, Bangor University, Wrexham, United Kingdom; 2 Welsh Institute of Physical Activity, Health and Sport, Swansea University, Swansea, United Kingdom

**Keywords:** Workplace, Evaluation, Mixed-methods, Mental well-being

## Abstract

**Background:**

Workplace physical activity interventions have shown positive outcomes for employee health, productivity and absenteeism (1,2). However, the majority prescribe the duration and/or type of activity to be undertaken. In response to strong public opinion that employers should do more to improve the health of their workforce, Public Health Wales, the public health agency for Wales, developed a 12-month pilot physical activity initiative - Time to Move (TTM). TTM allowed participants to use one hour/week (pro rata) of paid work time for any physical activity. We evaluated TTM to understand its impact and identify learning.

**Methods:**

Using pre-experimental time series design, data were collected from participating employees: baseline (June-August 2018), mid-initiative (December 2018) and at 12-months (June-August 2019). Using validated scales where possible, questionnaires recorded: physical activity (MET-minutes/week), general health (0, poor-100, good), mental well-being (SWEMWBS), job satisfaction (1, very dissatisfied-5, very satisfied) and demographics. Biometric measures (baseline, 12-months) included Body Mass Index (BMI) and blood pressure. Analyses used descriptive statistics, bivariate analysis and generalized linear modelling. Focus groups explored participants' perceptions of TTM, analysed thematically.

**Results:**

542 participants completed all measures (63.1% of baseline). Compared to baseline, at 12-months 57.7% reported increased physical activity (30.6% decreased; 11.6% no change) with 75.3% meeting UK activity guidelines (58.8% baseline). Those with the lowest levels of physical activity at baseline (n = 223) increased their weekly moderate activity by > 2.5 hours, whilst those with moderate activity (n = 269) increased by 58 minutes/week. A small improvement was reported in mental well-being (mean scores; 22.4 baseline, 23.2 12-months), with participants with low mental well-being at baseline improving the most. Self-reported health and job satisfaction also improved. However, BMI and blood pressure changes were non-meaningful. Employee attitudes to TTM were positive. Organisational support was a motivating factor for participation, with competing demands a barrier.

**Conclusions:**

The provision of paid time to engage in physical activity can improve employee health and well-being. TTM provides an example of how organisations can promote physical activity and change workplace culture. However, further research should explore the long-term impact of the intervention, including the potential impact of COVID-19-related restrictions.

